# GFP::PCN-1 does not reliably mark S phase in *C. elegans* adult germline progenitor zone cells

**DOI:** 10.17912/W20W9G

**Published:** 2018-05-30

**Authors:** Tokiko Uruta, Swathi Arur

**Affiliations:** 1 Department of Genetics, UT MD Anderson Cancer Center, Houston, TX, 77030

**Figure 1:**
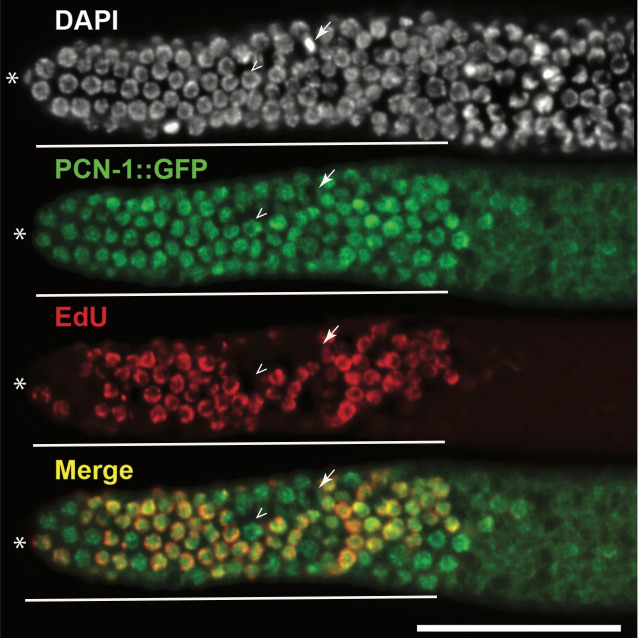
The figure shows an adult dissected hermaphroditic germline oriented with distal tip cell (*) on the left. The germline is stained with anti-GFP antibody, to visualize GFP::PCN-1 (green), DAPI to visualize the DNA (white) and EdU to visualize S phase cells (red). The solid line marks the progenitor zone. Arrowhead marks an example of non-EdU incorporating cell, with nuclear GFP::PCN-1. Arrow marks an example of non-nuclear GFP::PCN-1 in metaphase cells. Scale bar: 50μm.

## Description

PCNA (proliferating cell nuclear antigen) is the DNA polymerase processivity factor that loads onto the chromatin during S phase of the cell-cycle (Brauchle *et al.* 2003). Thus, nuclear localization of PCNA (PCN-1 in *C. elegans*) is used as a marker for the S phase of the cell cycle (Brauchle *et al.* 2003). GFP::PCN-1 has been shown to label S phase in *C. elegans* embryo when driven through the germline and embryonic promoter *pie-1* (Brauchle et al. 2003*)*. We assayed GFP::PCN-1 (allele *isIs17*, GZ264 (Brauchle *et al.* 2003)) as a marker for S phase in adult germline progenitor zone cells. If this reagent were a faithful marker of S phase in germline progenitor zone cells, we would expect nuclear localization during S phase, and nuclear exclusion in the other phases of the cell-cycle, as is the case in the *C. elegans* embryo. We would also expect a perfect overlap with EdU which marks S phase of the cell cycle. EdU is incorporated in ~55-60% of the adult hermaphroditic wild-type progenitor zone cells (Fox *et al.* 2011; Furuta *et al.* 2018). We found that GFP::PCN-1 was nuclear in almost all of the progenitor zone cells, irrespective of whether they were EdU positive or EdU negative (arrowhead, Figure 1). The only cells that excluded GFP::PCN-1 from the nucleus were the metaphase cells (arrow) during M phase, when the nuclear envelope breaks down. Thus, GFP::PCN-1 does not overlap with EdU labeling in adult progenitor zone cells. These data suggest that nuclear localization of GFP::PCN-1 is not a good marker of S phase dynamics in the *C. elegans* adult germline progenitor cells. This could either be because GFP signal perdures in the nucleus in this context, or that GFP::PCN-1 is nuclear localized throughout the cell cycle in germline progenitor zone cells, unlike in the *C. elegans* embryo.

## Reagents

GFP::PCN-1 worms were grown on nematode growth medium (NGM) plates with *E. coli* OP50 bacteria. Adult 24 hours past mid-L4 hermaphroditic worms were incubated with 200 µM of EdU solution for 10 minutes at room temperature in the dark. After the EdU soaking, the animals were dissected and extruded germlines processed using the Click-iT® Plus EdU Alexa Fluor® 594 Imaging Kit (ThermoFisher Scientific, catalog number C10639) per the manufacturer’s recommendations (Furuta *et al.* 2018). After EdU processing, the germlines were treated with anti-GFP antibody (developed in the Arur Lab), to visualize GFP::PCN-1, followed by treatment with donkey-anti-Rabbit-488 secondary antibody (Thermofisher Scientific, catalog number A21260). Antibody treated germlines were stained with 2μg/ml DAPI (4′,6-diamidine-2-phenylindole dihydrochloride) and finally suspended in 10μl of Vectashield (antifade agent).
